# The Prescription Pattern of Heart Failure Medications in Reduced, Mildly Reduced, and Preserved Ejection Fractions

**DOI:** 10.3390/jcm12010099

**Published:** 2022-12-22

**Authors:** Tripti Rastogi, Kevin Duarte, Olivier Huttin, François Roubille, Nicolas Girerd

**Affiliations:** 1 Centre d’Investigation Clinique Pierre Drouin—INSERM—CHRU de Nancy, Institut Lorrain du Coeur et des Vaisseaux Louis Mathieu, 54000 Nancy, France; 2 Cardiology Department, CHU de Montpellier, PhyMedExp, Université de Montpellier, INSERM, CNRS, 34090 Montpellier, France

**Keywords:** heart failure, therapeutic inertia, cardiovascular diseases, prescription score, cardio-renal syndrome

## Abstract

A substantial proportion of patients with heart failure (HF) receive suboptimal guideline-recommended therapy. We aimed to identify the factors leading to suboptimal drug prescription in HF and according to HF phenotypes. This retrospective, single-centre observational cohort study included 702 patients admitted for worsening HF (HF with a reduced ejection fraction [HFrEF], *n* = 198; HF with a mildly reduced EF [HFmrEF], *n* = 122; and HF with a preserved EF [HFpEF], *n* = 382). A score based on the prescription and dose percentage of ACEi/ARBs, β-blockers, and MRAs at discharge was calculated (a total score ranging from zero to six). Approximately 70% of patients received ACEi/ARBs/ARNi, 80% of patients received β-blockers, and 20% received MRAs. The mean HF drug dose was approximately 50% of the recommended dose, irrespective of the HF phenotype. Ischaemic heart disease was associated with a higher prescription score (ranging from 0.4 to 1) compared to no history of ischaemic heart disease, irrespective of the left ventricular EF (LVEF) level. A lower prescription score was associated with older age and male sex in HFrEF and diabetes in HFmrEF. The overall ability of the models to predict the optimal drug dose, including key HF variables (including natriuretic peptides at admission), was poor (R^2^ < 0.25). A higher prescription score was associated with a lower risk of re-hospitalization and death (HR: 0.75 (0.57–0.97), *p* = 0.03), irrespective of phenotype (*p*-interaction = 0.41). Despite very different HF management guidelines according to LVEF, the prescription pattern of HF drugs is poorly related to LVEF and clinical characteristics, thus suggesting that physician-driven factors may be involved in the setting of therapeutic inertia. It may also be related to drug intolerance or clinical stability that is not predicted by the patients’ profiles.

## 1. Background

The use of ACE inhibitor/angiotensin receptor blockers (ACEi/ARBs), β-blockers, and mineralocorticoid receptor antagonists (MRAs) is known to improve prognoses in heart failure (HF) with a reduced ejection fraction (HFrEF) and a mildly reduced ejection fraction (HFmrEF). Current guidelines subdivide HF phenotypes into three categories based on the ejection fraction: HFrEF, HFmrEF, and HF with a preserved ejection fraction (HFpEF) [1]. Guidelines have recommended the highest tolerated dose of drug treatment for HFrEF for years and have recently extended this approach to HFmrEF for ACEi/ARBs, β-blockers, and MRAs [1,2]. Although recent studies show that the majority of patients with HFrEF are prescribed ACEi/ARBs (ranging from 60% to 89%) and β-blockers (ranging from 67% to 88%), only a small proportion are prescribed the target dose in community settings (17% to 54%) [3,4,5,6] with lower doses possibly partially related to therapeutic inertia [7]. A number of factors impact drug prescription, including age, comorbidities, and particularly renal function. Moreover, the level of evidence is lower in patients with LVEF > 40% and may influence the decision on drug dosage. Previous studies have implemented prescription adherence scores (based on the proportion of drug dose according to guideline-recommended therapy in HFrEF) and examined the baseline factors associated with guideline-recommended therapy and its impact on cardiovascular outcomes [8,9]. However, there are limited data as to which clinical variables influence the prescription patterns leading to HF drug prescription [10].

## 2. Aim

The aims of this study were to identify (i) the factors associated with the prescription and higher dosage of ACEi/ARBs, β-blockers, and MRAs according to HF phenotypes (HFrEF, HFmrEF, and HFpEF) and (ii) the association between cardiovascular outcome (the first re-hospitalization for HF or death) and higher prescription score according to the HF phenotype.

## 3. Methods

### 3.1. Study Design and Population

This retrospective, observational study included 741 patients admitted for worsening HF between January 2015 and May 2019 in the cardiology department of a tertiary care teaching hospital in Nancy, France. Patients were identified using an integrated clinical and research database according to specific coding based on the international classification of disease (ICD-9) codes related to HF. Patients were included if they were older than 18 years of age and diagnosed with AHF regardless of their aetiology and systolic function and fulfilled the European society of cardiology criteria [11]. Patients with severe valvular heart disease, post-operative valve replacement, congenital cardiomyopathy, and the absence of available echocardiographic data during hospitalization were excluded (*n* = 39). The corroboration of the diagnosis of HFpEF was obtained by calculating the weighted composite H2FPEF score as described by Reddy et al. [12].

### 3.2. Data Collection and Storage

Data were collected by cardiology interns and dedicated staff and recorded in Microsoft Excel^®^ files. The data were then processed by the data management team at the Clinical Investigation Centre, Nancy and used after the resolution of queries.

### 3.3. Statistical Analysis

For the descriptive analyses, continuous variables are expressed as the mean  ±  standard deviation (SD) for the normally distributed data or as the median (Q1–Q3) for the skewed data. The categorical variables are expressed as proportions (%). The normality of the distribution of the continuous variables was verified by plotting histograms and QQ plots. The baseline characteristics were compared using an ANOVA or the Kruskal-Wallis test for the continuous variables, as appropriate, and Fisher’s exact test or the chi-square test for the categorical variables.

A score evaluating the prescription of the guideline-recommended therapy of ACEi, ARBs, β-blockers, and MRAs at the time of discharge in patients with HF was adapted from the adherence score described by Komajda et al. [8]. The score was calculated via the summation of points attributed according to the dose of each drug class. The points were attributed as follows: 0 points for non-prescription, 1 point for a prescription at less than 50% of the recommended target dose, and 2 points for a prescription at 50% or above the recommended dose (according to the ESC 2021/AHA 2017 guidelines). For β-blockers, if the drug prescribed was not a recommended β-blocker for HFrEF (i.e., metoprolol, carvedilol, bisoprolol, and nebivolol), 1 point was attributed. The scores according to the drug class are detailed in Table 1.

In order to identify the factors associated with a significant increase in the prescription score and to assess the magnitude of change in the prescription score according to the clinical variables, multiple linear regressions were performed with the clinical variables as covariates (assessed at the time of admission) and the total score as outcome (a variable ranging from 0 to 6) in patients with HFrEF, HFmrEF, and HFpEF. The clinical covariates entered in the models were: the age at hospitalization, sex, BMI, history of ischaemic heart disease (IHD), hypertension, diabetes, smoking or past-smoking status, alcohol intake, BNP, heart rate, systolic blood pressure (SBP), haemoglobin, NYHA class, and eGFR at the time of admission. The BNPs were converted to logarithmic values due to non-linearity. A stepwise backward selection approach was used whilst adjusting for age and sex to assess the clinical variables associated with a higher prescription score in each HF phenotype.

The patients in each HF phenotype were then subdivided according to higher (≥4) and lower (<4) prescription scores. Kaplan-Meier curves were subsequently plotted to estimate the survival probabilities in patients having higher (≥4) and lower (<4) HF drug prescription scores in each HF phenotype. In addition, the risk of association between the cardiovascular outcome and a higher (≥4) prescription score (with a lower prescription score (<4) used as the reference group) was assessed using Cox proportional hazard ratios (model 1: adjusted for age and sex, and model 2: adjusted for age at hospitalization, sex, BMI, IHD, hypertension, diabetes, eGFR, NYHA category, and diuretic therapy). The association between the clinical variables as covariates and total scores as the outcome was also assessed using ordinal regression.

A two-tailed *p* value < 0.05 was considered statistically significant. All analyses were performed using R statistical software (version 4.0.5) (R Core Team (2020). R: A language and environment for statistical computing. R Foundation for Statistical Computing, Vienna, Austria. URL: https://www.R-project.org/.). Missing data were not inputted in the analyses.

This single-centre study comprised data gathered from hospital charts and, per French law, was declared to the National Commission for Information Technology and Civil Liberties (CNIL) and did not require individual patient consent.

## 4. Results

### 4.1. Baseline Characteristics

Among the 702 study participants included in this analysis, 198 (28%) patients had HFrEF, 122 (17%) had HFmrEF, and 382 (54%) had HFpEF (Table 2).

**Figure 1:**
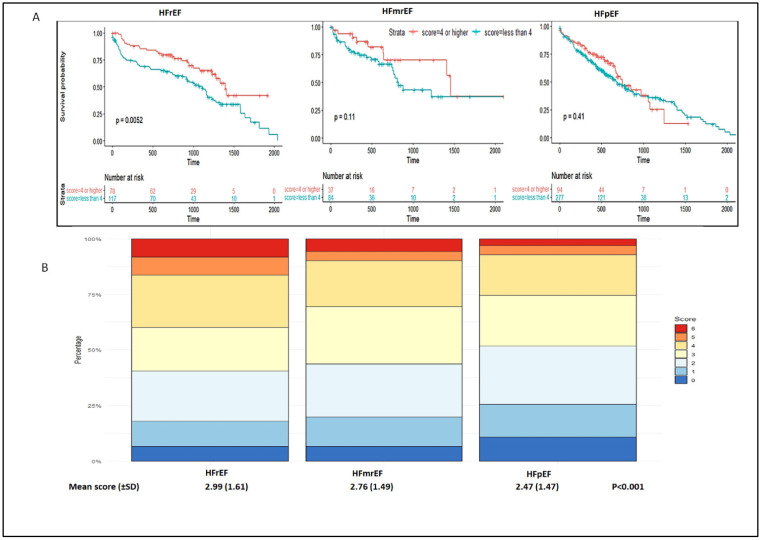
Survival plots according to the prescription score of HF drugs in HFrEF, HFmrEF, and HFpEF. (**A**) Survival plots; (**B**) prescription score for each phenotype. In panel B, the y-axis signifies the percentage of patients.

The patients with HFrEF were more likely to be male and younger (mean age: 71.4 ± 13.1 years) compared to patients with HFmrEF and HFpEF (75.3 ± 12.6 and 78.2 ± 11.1 years, respectively). IHD was more frequently observed in HFrEF (HFrEF 59.7% vs. HFmrEF 42.5% vs. HFpEF 35.9%; *p* < 0.0001), whereas hypertension and diabetes were more commonly observed in HFpEF (hypertension: HFpEF 88.0% vs. HFmrEF 82.8% vs. HFrEF 68.7%; *p* < 0.001; diabetes: HFpEF 41.6% vs. HFrEF 33.8% vs. HFmrEF 30.3%, *p* = 0.037). A total score of four points or above (any two drugs prescribed at a higher dose or all three drugs prescribed with at least one at a higher dose) was observed in approximately 40.0%, 30.6%, and 25.3% of patients with HFrEF, HFmrEF, and HFpEF, respectively (Table 1 and Figure 1B). The median [IQR] H2FPEF score was five [4,5,6,7] in patients for whom all variables were available (Table 3).

Overall, ACEi/ARBs/ARNi were prescribed in 80%, 77%, and 69% of patients with HFrEF, HFmrEF, and HFpEF, respectively, whereas ß-blockers were prescribed in 88%, 81%, and 75% of patients with HFrEF, HFmrEF, and HFpEF, respectively. However, MRAs were only prescribed in 28%, 14%, and 21% of patients with HFrEF, HFmrEF, and HFpEF, respectively. The mean dose of ACEi/ARBs, β-blockers, and MRAs was approximately 50% of the recommended dose (Table 4).

### 4.2. Association between the Clinical Variables and HF Drug Dose

In all three HF phenotypes, a history of IHD was associated with an approximately 0.4 to 1 unit increase in the prescription score (HFrEF β = 0.86 (0.37–1.35) *p* = 0.0007; HFmrEF β = 1.02 (0.45–1.56) *p* = 0.0003; HFpEF β = 0.43 (0.11–0.75) *p* = 0.009) (Table 3).

In patients with HFrEF, the regression model minimally explained the total score obtained for the guideline-recommended treatment (adjusted R^2^ = 0.16). Older age and male sex were associated with lower scores, whereas better renal function was associated with higher prescription scores (Table 5).

In patients with HFmrEF, smoking or past smoking was associated with a 0.78 unit increase in the total score (β = 0.78 (0.16–1.39), *p* = 0.014), whereas diabetes was associated with a 0.79 unit decrease in the total score (β = −0.79 (−1.38–−0.20), *p* = 0.009) (adjusted R^2^ = 0.22). A higher BMI and SBP were also associated with higher prescription scores.

In patients with HFpEF, the clinical variables minimally explained the prescription pattern (adjusted R^2^ = 0.11). The total score increased by 0.9 units in patients with hypertension (β = 0.98 (0.47–1.46), *p* = 0.001) and by 0.1 unit per 10 mL/min/1.73 m^2^ increase in the eGFR (β = 0.1 (0.04–0.16); *p* = 0.002).

The results did not differ from those obtained with the linear regression when performing an ordinal regression as a sensitivity analysis (Supplementary Table S1).

### 4.3. Risk of Hospitalization for HF or Death According to the Total Score

Over a median follow-up of 17.1 [6.3–29.0] months, there were 346 (49.2%) patients either re-admitted for HF or deceased (150 patients re-admitted and 261 patients deceased). Overall, a higher total score was associated with a lower risk of re-hospitalization and death (HR: 0.75 (0.57–0.97), *p* = 0.03, *p*-interaction: 0.41; HFrEF HR: 0.63 (0.40–1.00), *p* = 0.049, HFmrEF HR: 0.63 (0.30–1.34), *p* = 0.23 and HFpEF HR: 0.94 (0.66–1.33), *p* = 0.72) in model 1 (adjusted for age and sex). Patients with HFrEF having a higher total score had a greater survival probability compared to patients having a lower total score (*p* = 0.005) (Figure 1A). However, in the fully adjusted model (model 2), the overall risk of mortality did not differ between patients with higher and lower scores (*p* for interaction = 0.74).

## 5. Discussion

In this study, over 70% of patients received ACEi/ARBs individually, and over 80% of patients received β-blockers individually, while very few patients were prescribed MRAs irrespective of their HF phenotype. The mean dose of all three drugs was approximately 50% regardless of their HFrEF, HFmrEF, and HFpEF status. Overall, a higher score was associated with a lower risk of re-hospitalization for HF and death. The fact that the HF drug treatment did not largely differ across LVEF strata was unexpected, although in line with previous studies [6,13]. It is possible that the concomitant comorbidities (IHD and hypertension) were the main triggers of ACEi and β-blocker initiation in patients with HFpEF.

### 5.1. Baseline Characteristics and Prescription Pattern of the Drug Class and Target Dose

The baseline characteristics of patients with HF were similar to previously published studies [4,5]. Patients with HFrEF were more likely to be younger and have ischaemic cardiovascular diseases, and less likely to have hypertension compared to patients with HFpEF [4,5]. However, diabetes was observed more frequently in patients with HFpEF compared to HFrEF and HFmrEF (*p* = 0.037) in the present study, whereas in the CHARM and ESC-HF long-term registry cohorts, the proportion of patients with diabetes was similar across all three phenotypes (*p* = 0.14 and *p* = 0.71, respectively) [14,15].

The prescription rate of RAS inhibitors in patients with HFrEF (80%) in our study was similar to the prescription rate of RAS inhibitors (78%) in the OFICA study (a single-day survey of patients with HF at discharge) conducted across 170 hospitals in France in 2009. However, the prescription rate of β-blockers (88%) was higher in our study compared to that of β-blockers (67%) in the OFICA study [16]. Importantly, the prescription rate of MRAs both herein and in the OFICA study remained similarly low (28% vs. 26%) despite the temporal difference in the two studies. A previously published study by Uijl et al. on the temporal trends of HF drug prescription rates also reported similar findings [16]. Uijl et al. noted that the prescription rate for β-blockers increased (30% to 55%) but remained low for MRAs (20%) over a span of 13 years in patients after being diagnosed with HF [16]. Given that MRAs are contraindicated in severe renal dysfunction, the underuse of MRAs was associated with poorer renal function [17]. However, in the current analysis, more than 45% of patients with HFrEF and HFmrEF had an eGFR ≥ 60 mL/min/1.73 m^2^ suggesting the continued underuse of MRAs.

Despite the increase in the prescription rate of ACEi/ARBs and β-blockers, the prescription of higher dosages (≥50% of the target dose) remains low. Experts suggest that this lower-dose HF therapy could be partially explained by the “risk treatment paradox” whereby sicker patients are prescribed fewer medications at a lower dose and because stable symptoms in HF are misinterpreted as low-risk HF [7,18]. However, a recent study suggests that lower adherence to guideline-directed medical therapy may be related to limiting physiological factors and comorbidities [19].

### 5.2. Factors Associated with Suboptimal Prescription Scores

In the present study, IHD was consistently associated with a higher prescription score irrespective of HF phenotype. These findings are consistent with a previous study in which IHD was found to be a positive predictor of β-blocker prescription in patients with HFrEF and HFpEF [5].

In HFrEF, older age and male sex were associated with lower scores and are partially in line with previous studies, whereby older age was associated with lower adherence to guideline-recommended therapy in HFrEF [5,19]. Moreover, hypertension was associated with higher odds of prescription of HF therapy in patients with HFpEF. In our study, the heart rate, NYHA class, and BNP level at the time of admission did not appear to influence the prescription scores in any of the HF phenotypes, whereas in the previously reported literature, higher NYHA classes and lower HRs were associated with a lower prescription of HF therapy [19]. In addition, diabetes was found herein to be associated with lower prescription scores in patients with HFmrEF but not with other HF phenotypes, while a previous study reported that diabetes was associated with higher adherence to guideline-recommended therapy in HFrEF [19]. Furthermore, the conventional risk factors for low-dose HF therapy, such as female sex and low blood pressure [20], were not related to drug dose in the present analysis. Our results are similar to those of a multinational population-based registry where hypotension was not associated with lower drug doses, although chronic kidney disease was associated with the prescription of lower doses of ACEi/ARBs but not of β-blockers [21]. The discrepancy between our findings and other reports could be due to differences in the baseline characteristics of the studied patient populations since our analysis was conducted in hospitalized patients who had a higher NYHA III/IV and higher mean HR compared to the two aforementioned studies in ambulatory patients. In addition, better renal function was associated with marginally higher scores in both HFrEF and HFpEF patients, which is consistent with previous studies.

Most importantly, the ability of the clinical characteristics to predict HF drug dose was low (adjusted R^2^ ≤ 0.25), albeit similar to a previously published study assessing prescription adherence according to clinical variables [5], thus suggesting that factors other than clinical characteristics are involved in prescription patterns. The latter could potentially be related to drug intolerance not predicted by the patient’s profile. Furthermore, the hemodynamic stability, HF progression, and prescription of concomitant medication for comorbidities may impact the drug dosage or lead to the discontinuation of the drug [5,22]. Among patients with HFrEF, 18% had COPD, which may have resulted in the prescription of lower β-blocker doses. In addition, very few of our study patients had hypotension, and the mean heart rate was above 88 bpm; nevertheless, β-blockers remained under-prescribed in patients with HFrEF, although such under-prescription may potentially be physician-driven in the setting of therapeutic inertia [7,23]. Further, recent studies have suggested that in addition to HF drugs, cardiac resynchronization therapy may affect long-term outcomes in patients with advanced HF [24,25]. However, very few patients in our study had cardiac resynchronization therapy at the time of discharge. Hence, the impact of this aforementioned therapy could not be examined.

There are several limitations to our study. This is a single-centre study, and thus the results may not be generalizable to other cohorts. Contra-indication and drug intolerance information could not be included in the calculation of the prescription score due to unavailable data. In addition, in-hospital medication changes could not be analyzed due to the lack of medication data at the time of admission. The BNP levels at discharge were available for only a limited number of patients and thus could not be included in the analysis. Weak evidence for HF medication in HFpEF patients could have impacted the prescription (score) calculation; however, HF drug prescription patterns were surprisingly unrelated to LVEF despite striking differences in the level of evidence. Moreover, LVEF changes over time could have resulted in an overestimation of underuse in patients with HFrEF and HFmrEF who previously had a preserved ejection fraction. The study enrolment period (2015 to 2019) was conducted prior to the ESC 2021 and AHA 2017 guidelines, which may have influenced the prescription pattern in HFmrEF and the prescription of ARNi in HFmrEF. Furthermore, the (most) recent prescription score [26] could not be applied since few patients were prescribed ARNi and ivabradine, while SGLT2i was not indicated for HF during the study period. Importantly, LVEF is not the sole echocardiographic variable that should be considered in HF. Specifically, taking into account right ventricular function (p.e. with TAPSE) is of interest but unrelated to the guidelines of drug prescription in HF.

## 6. Conclusions

The prescription pattern of HF drugs was unrelated to the ejection fraction and poorly related to the clinical phenotype, suggesting that physician-driven factors may be involved in the setting of therapeutic inertia. Other factors, such as clinical stability and polypharmacy, not recorded in this study, may also impact the prescription pattern of HF medication.

## Figures and Tables

**Table 1:** Number of patients in each drug class according to the drug class and score.

Drug Class	Score	HFrEF	HFmrEF	HFpEF
ACEi/ARBs	0	58 (29.2)	33 (27.0)	143 (37.4)
	1	56 (28.3)	28 (23.0)	86 (22.5)
	2	82 (41.4)	60 (49.2)	144 (37.7)
β-blockers	0	22 (11.1)	22 (18.0)	92 (24.1)
	1	84 (42.4)	45 (36.9)	146 (38.2)
	2	89 (44.9)	54 (44.3)	136 (35.6)
MRA	0	139 (70.2)	103 (84.4)	298 (78.0)
	1	9 (4.5)	3 (2.5)	11 (2.9)
	2	47 (23.7)	15 (12.3)	64 (16.8)

ACEi/ARBs: ACE inhibitor/angiotensin receptor blockers; MRAs: mineralocorticoid receptor antagonists; HFrEF: heart failure with reduced ejection fraction; HFmrEF: heart failure with mildely reduced ejection fraction; HFpEF: heart failure with preserved ejection fraction.

**Table 2:** Baseline patient characteristics according to HF phenotype ^§^.

Variable	*n* Available	Overall	*n*	HFrEF	*n*	HFmrEF	*n*	HFpEF	*p*
		*n* = 702		*n* = 198		*n* = 122		*n* = 382	
Clinical characteristics									
Age at hospitalisation (y)	702	75.8 (12.3)	198	71.4 (13.1)	122	75.3 (12.6)	382	78.2 (11.1)	<0.001
Male sex	702	350 (49.9)	198	120 (60.6)	122	65 (53.3)	382	165 (43.2)	<0.001
BMI	678	27.0 (5.9)	188	25.9 (5.2)	116	26.9 (5.8)	374	27.6 (6.3)	0.009
Smoking/past-smoking (%)	702	217 (30.9)	198	68 (34.3)	122	36 (29.5)	382	113 (29.6)	0.467
Alcohol intake (%)	702	64 (9.1)	198	23 (11.6)	122	9 (7.4)	382	32 (8.4)	0.334
Ischemic heart disease (%)	621	267 (43.0)	154	92 (59.7)	113	48 (42.5)	354	127 (35.9)	<0.001
STEMI (%)	702	125 (17.8)	198	53 (26.8)	122	22 (18.0)	382	50 (13.1)	<0.001
Hypertension (%)	702	573 (81.6)	198	136 (68.7)	122	101 (82.8)	382	336 (88.0)	<0.001
Diabetes (%)	702	263 (37.5)	198	67 (33.8)	122	37 (30.3)	382	159 (41.6)	0.037
QRS > 120 (%)	702	187 (26.7)	196	56 (28.6)	122	37 (30.3)	382	94 (24.6)	0.363
Atrial fibrillation	691	267 (38.6)	195	58 (29.7)	119	55 (46.2)	377	154 (40.8)	0.006
LVEF (mean (SD))	702	49.01 (15.7)	198	28.3 (7.5)	122	44.9 (2.6)	382	61.0 (6.9)	<0.001
Heart rate	701	87.12 (24.56)	198	88.32 (23.37)	122	93.02 (27.58)	381	84.61 (23.82)	0.003
SBP	701	138.30 (32.4)	198	122.3 (24.2)	122	139.1 (30.6)	381	146.4 (33.6)	<0.001
Hypotension (BP < 100)		69 (9.8)		34 (17.2)		13 (10.7)		22 (5.8)	<0.001
COPD	499	90 (18.0)	84	13 (15.5)	92	14 (15.2)	323	63 (19.5)	0.512
Chronic respiratory insufficiency	499	90 (18.0)	84	2 (2.4)	92	15 (16.3)	323	73 (22.6)	<0.001
Crepitation	499	437 (87.6)	84	62 (73.8)	92	81 (88.0)	323	294 (91.0)	<0.001
Leg oedema	702	456 (65.0)	198	113 (57.1)	192	78 (63.9)	382	265 (69.4)	0.013
NYHA (%)	702		198		122		382		<0.001
Class I		5 (0.7)		3 (1.5)		2 (1.6)		0 (0.0)	
Class II		41 (5.8)		23 (11.6)		6 (4.9)		12 (3.1)	
Class III		292 (41.6)		83 (41.9)		45 (36.9)		164 (42.9)	
Class IV		364 (51.9)		89 (44.9)		69 (56.6)		206 (53.9)	
Laboratory investigations									
Haemoglobin	693	12.3 (2.2)	195	12.88 (2.2)	120	12.4 (2.2)	378	11.9 (2.1)	<0.001
BNP at admission	626	746 [421–1469]	166	1218 [611–2182]	109	799 [538–1460]	351	583 [352–1024]	<0.001
eGFR (MDRD)	691	54.6 (24.4)	193	58.2 (24.1)	121	57.4 (25.1)	377	51.9 (24.1)	0.006
≥60		273 (39.5)		91 (47.2)		55 (45.5)		127 (33.7)	0.011
30–60		301 (43.6)		78 (40.4)		47 (38.8)		176 (46.7)	
<30		117 (16.9)		24 (12.4)		19 (15.7)		74 (19.6)	

BMI: body mass index; BNP: brain natriuretic peptide; eGFR: estimated glomerular filtration rate; SBP: systolic blood pressure; STEMI: ST-segment elevation myocardial infarction; MDRD: modification of diet in renal disease. Alcohol intake and smoking were patient-reported variables. ^§^ The baseline characteristics were assessed at the time of admission, whereas the GDMT score was calculated according to the dose and prescription at the time of discharge.

**Table 3:** H2FPEF score in patients diagnosed with HFpEF.

Score	Probability of HFpEF	Number
0–1	Low	5 (1.5%)
2–5	Intermediate	183 (55.3%)
6 and above	High	143 (43.2%)

**Table 4:** Specifics of HF drug prescriptions according to HF phenotype ^§^.

Variable	*n* Available	Overall	*n*	HFrEF	*n*	HFmrEF	*n*	HFpEF	*p*
		*n* = 702		*n* = 198		*n* = 122		*n* = 382	
Drug class									
ACEi/ARB/ARNi	692	510 (73.7)	196	157 (80.1)	121	94 (77.7)	375	259 (69.1)	0.01
ACEi (%)	690	363 (52.6)	196	123 (62.8)	121	71 (58.7)	373	169 (45.3)	<0.001
ARB (%)	688	141 (20.5)	196	18 (9.2)	121	26 (21.5)	371	97 (26.1)	<0.001
ARNi (%)	689	21 (3.0)	196	16 (8.2)	121	2 (1.7)	312	3 (0.8)	<0.001
β-blocker (%)	690	554 (80.3)	195	173 (88.7)	121	99 (81.8)	374	282 (75.4)	0.001
MRA (%)	689	149 (21.6)	195	56 (28.7)	121	18 (14.9)	373	75 (20.1)	0.008
Loop diuretics (%)	689	578 (83.9)	195	156 (80.0)	121	99 (81.8)	373	323 (86.6)	0.101
Ivabradine (%)	404	7 (1.7)	195	4 (2.1)	69	1 (1.4)	140	2 (1.4)	0.894
β-blocker recommended * (%)	554	470 (84.8)	195	165 (95.4)	99	82 (82.8)	282	223 (79.1)	<0.001
ACEi/ARB dose %	489	50.9 (31.1)	141	49.56 (29.56)	93	54.7 (32.8)	255	50.2 (31.4)	0.412
ACEi/ARB dose % for the whole cohort ^#^	690	36.1 (34.9)	196	35.66 (33.55)	121	42.1 (36.9)	373	34.3 (34.9)	0.105
β-blocker dose %	470	49.4 (28.1)	165	41.11 (29.79)	82	42.3 (33.9)	223	35.3 (32.8)	0.057
β-blocker dose % for the whole cohort ^#^	606	38.3 (32.2)	187	46.59 (27.38)	104	53.7 (29.1)	315	49. 9 (28.1)	0.165
MRA dose %	149	56.9 (28.8)	56	54.02 (22.25)	18	51.4 (20.1)	75	60.3 (34.2)	0.320
MRA dose % for the whole cohort ^#^	689	12.3 (26.9)	195	15.51 (27.22)	121	7.6 (19.8)	373	12.1 (28.6)	0.041
Total score≥ 4 (%)	687	209 (30.4)	195	78 (40.0)	121	37 (30.6)	371	94 (25.3)	0.002

ACEi: ACE inhibitor; ARB: angiotensin receptor blocker; ARNi: angiotensin receptor blocker and neprilysin inhibitor; MRA: mineralocorticoid receptor antagonist. ^§^ The GDMT score was calculated according to the dose and prescription at the time of discharge. * For β-blockers, the drug was a recommended β-blocker for HFrEF if it was mentioned as a recommended β-blocker in the guidelines (i.e., metoprolol, carvedilol, bisoprolol, and nebivolol). ^#^ The dose for the whole cohort included those not receiving the drug and was recorded as receiving zero doses of the drug for the analysis.

**Table 5:** Predictors of HF drug prescription and higher prescription score in HFrEF, HFmrEF, and HFpEF using the linear regression model.

		HFrEF	Adjusted R^2^ = 0.16		HFmrEF	Adjusted R^2^ = 0.22		HFpEF	Adjusted R^2^ = 0.11
Variable	Estimate	95% CI	*p* Value	Estimate	95%CI	*p* Value	Estimate	95%CI	*p* Value
(Intercept)	4.50	(2.76–6.24)	<0.0001	−1.16	(−3.69–1.37)	0.365	1.1	(0.68–3.24)	0.003
Age at hospitalisation	−0.03	(−0.05–−0.01)	0.0008	0.01	(−0.01–0.03)	0.482	−0.01	(−0.03–0.00)	0.083
Male sex	−0.614	(−1.12–−0.11)	0.016	−0.25	(−0.81–0.31)	0.383	−0.20	(−0.53–0.13)	0.240
BMI		/		0.07	(0.02–0.11)	0.004		/	
Smoking/past smoking		/		0.78	(0.16–1.39)	0.014	0.39	(0.02–0.75)	0.039
Ischaemic heart disease	0.86	(0.37–1.35)	0.0007	1.02	(0.48–1.56)	0.0003	0.43	(0.11–0.75)	0.009
SBP at admission		/		0.01	(0.00–0.02)	0.039		/	
Hypertension		/			/		0.98	(0.47–1.46)	0.0001
Diabetes		/		−0.79	(−1.38–−0.20)	0.009		/	
eGFR (MDRD)/10 mL/min/1.73 m^2^	0.12	(0.02–0.22)	0.022		/		0.10	(0.04–0.16)	0.002

logBNP, haemoglobin, alcohol intake and heart rate at admission were not retained as predictors of the prescription score for any of the HF phenotypes. BMI: body mass index; BNP: brain natriuretic peptide; eGFR: estimated glomerular filtration rate; SBP: systolic blood pressure.

## Data Availability

Data is available upon reasonable request to the study coordonator (N.G.).

## Supplementary Materials

The following supporting information can be downloaded at: https://www.mdpi.com/article/10.3390/jcm12010099/s1, Table S1: Predictors of prescription of HF therapy and higher dose in HFrEF, HFmrEF and HFpEF using ordinal regression model.
