# Supplementary material for: The Prescription Pattern of Heart Failure Medications in Reduced, Mildly Reduced, and Preserved Ejection Fractions
Source: J Clin Med. 2022 Dec 22;12(1):99. doi: 10.3390/jcm12010099 (PMC9821188; doi:10.3390/jcm12010099)
Supplement: Supplementary file 1 [file jcm-12-00099-s001.zip › jcm-2067742-supplementary.pdf]

Journal of
Clinical Medicine

MDPI

Supplementary Table S1. Predictors of prescription of HF therapy and higher dose in HFrEF, HFmrEF and HFpEF using ordinal regression model.

HFrEF	AIC:	526			HFmrEF	AIC:	367		HFpEF	AIC:	1138	
	OR	95%CI		p	OR	95%CI		p	OR	95%CI		p
Age	0.98	(0.96 -	0.99)	<0.01	1.01	(0.99 -	1.02)	0.44	0.98	(0.97 -	1.00)	0.24
Sex = male	0.63	(0.44 -	0.90)	0.01	0.82	(0.53 -	1.27)	0.38	0.99	(0.98 -	1.00)	0.15
IHD	1.88	(1.31 -	2.69)	<0.01	2.29	(1.48 -	3.56)	<0.01	1.44	(1.14 -	1.84)	<0.01
BMI		/			1.06	(1.02 -	1.09)	<0.01	1.02	(1.00 -	1.04)	0.05
Smoking/past-smoking		/			1.87	(1.15 -	3.03)	0.01	1.35	(1.03 -	1.77)	0.03
diabetes		/			0.52	(0.32 -	0.83)	<0.01		/		
eGFR change per 10ml	1.09	(1.02 -	1.17)	0.02		/			1.08	(1.03 -	1.14)	<0.01
SBP at admission		/			1.01	(1.00 -	1.01)	0.03		/		
Hypertension		/							1.97	(1.35 -	2.89)	<0.01
